# On the Robustness of Graph-Based Clustering to Random Network Alterations

**DOI:** 10.1074/mcp.RA120.002275

**Published:** 2020-11-24

**Authors:** R. Greg Stacey, Michael A. Skinnider, Leonard J. Foster

**Affiliations:** 1 Michael Smith Laboratories, University of British Columbia, Vancouver, Canada; 2 Department of Biochemistry, University of British Columbia, Vancouver, Canada

**Keywords:** Biochemistry, Bioinformatics, Proteomics, Interactome, Graph-based clustering, Robust, Noise, AP-MS, affinity purification-mass spectrometry, CO, ClusterONE, CORUM, comprehensive resource of mammalian complexes, GA, geometric accuracy, GO, Gene Ontology, MCL, Markov clustering, Y2H, yeast two-hybrid

## Abstract

Biological functions emerge from complex and dynamic networks of protein–protein interactions. Because these protein–protein interaction networks, or interactomes, represent pairwise connections within a hierarchically organized system, it is often useful to identify higher-order associations embedded within them, such as multimember protein complexes. Graph-based clustering techniques are widely used to accomplish this goal, and dozens of field-specific and general clustering algorithms exist. However, interactomes can be prone to errors, especially when inferred from high-throughput biochemical assays. Therefore, robustness to network-level noise is an important criterion. Here, we tested the robustness of a range of graph-based clustering algorithms in the presence of noise, including algorithms common across domains and those specific to protein networks. Strikingly, we found that all of the clustering algorithms tested here markedly amplified network-level noise. Randomly rewiring only 1% of network edges yielded more than a 50% change in clustering results. Moreover, we found the impact of network noise on individual clusters was not uniform: some clusters were consistently robust to injected noise, whereas others were not. Therefore we developed the *clust.perturb* R package and Shiny web application to measure the reproducibility of clusters by randomly perturbing the network. We show that *clust.perturb* results are predictive of real-world cluster stability: poorly reproducible clusters as identified by *clust.perturb* are significantly less likely to be reclustered across experiments. We conclude that graph-based clustering amplifies noise in protein interaction networks, but quantifying the robustness of a cluster to network noise can separate stable protein complexes from spurious associations.

## Highlights

• Metrics for measuring cluster similarity can be misleading and unintuitive.
• Using an intuitive metric, clustering is very sensitive to network noise.
• However, some clusters within a set are robust to network changes.
• *clust.perturb* predicts robust clusters by randomly perturbing the network.

## Graphical Abstract

**Figure:**
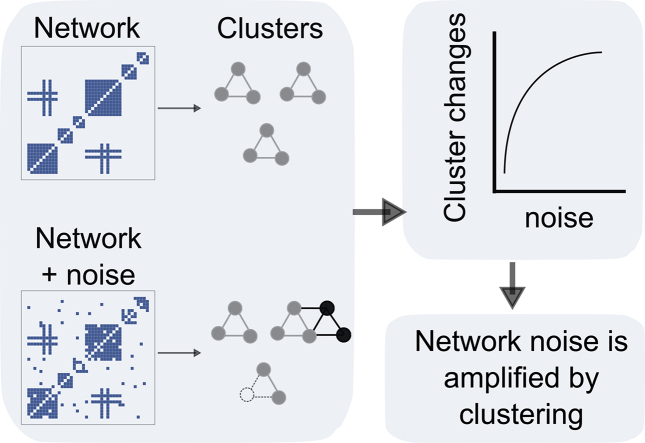

## In Brief

Networks are an important way to represent the connections within a system, and it is useful to identify tightly connected clusters within a network (*e.g.,* protein complexes). However, biological networks often have noise, and our results demonstrate that clustering is sensitive to noise at levels typical of biological networks (*e.g.,* 10–50%). We developed an R-based tool (*clust.perturb*) that can predict the real-world reproducibility of individual clusters for any clustering algorithm by randomly perturbing the network.

Networks are an important framework for representing the connections within a system, such as the agglomeration of proteins into complexes. Because these networks are composed of a list of pairwise connections (edges) between members (nodes) and do not explicitly detail higher-order associations, it can be useful to infer higher-order arrangements from the network. This task, called community detection or graph-based clustering, is ubiquitous across fields and is especially important in biology, where the function of a biological macromolecule such as a protein is often mediated by its interacting partners within the network.

However, noise in networks can complicate clustering. This is especially true in biological networks constructed from high-throughput experiments, such as protein–protein interaction networks (“interactomes”) where more than half of the expected network edges may vary from experiment to experiment, either because of errors in network reconstruction or changes in experimental conditions (1). Complicating this issue is the fact that it can be surprisingly ambiguous to measure differences between sets of clusters, in part because metrics for this purpose make different choices about how to penalize false positives (incorrectly merging clusters) *versus* false negatives (incorrectly separating clusters). This choice of weighting can mean popular metrics display biases and other nonintuitive behavior (2), and papers using these metrics can include in-depth discussions of their behavior (3). This may explain why there is some discrepancy in the literature regarding the degree of noise sensitivity when clustering biological networks, with some papers reporting substantial noise sensitivity (4) and others not (5, 6, 7).

The aim of the present study was to quantify the relationship between the level of network noise and cluster reproducibility. Our analysis focuses primarily on protein–protein interaction networks, but we reproduce our central findings in other types of networks. We first identified an unbiased cluster set similarity metric that behaved intuitively. With this metric in hand, we then randomly altered a “gold standard” unweighted network to varying degrees and measured the effects on the derived clusters. To arrive at general findings, we additionally analyzed two literature-curated interactomes (8, 9); three large-scale human interactomes derived from affinity purification–mass spectrometry (AP-MS) or yeast two-hybrid (Y2H) techniques, including one weighted interactome (10, 11, 12); a network of drug–drug side-effects (13); a representative social network (14, 15); and 28 protein–protein interaction networks derived from co-fractionation mass spectrometry experiments generated by our group (16, 17, 18, 19). We quantified the robustness of clusters obtained from nine clustering algorithms that are widely used in a number of different contexts. We found substantial sensitivity of clustering results to the injection of small amounts of noise into the networks. In particular, we found that all of the clustering algorithms tested here “amplified” noise in the network, such that a small perturbation of the underlying network produced a marked perturbation of the recovered complexes. We then reasoned that individual clusters that are robust to small perturbations are more likely to represent biologically or sociologically coherent communities. To this end, we developed the tool *clust.perturb*, an R package (https://github.com/fosterlab/clust-perturb) and the Shiny web application (https://rstacey.shinyapps.io/clust-perturb-tool/), that measures the reproducibility of clusters, and nodes within clusters, over multiple iterations of network perturbation. We show that *clust.perturb* can accurately predict which clusters are likely to be reproduced in real-world situations where networks vary (experiment-to-experiment changes), motivating its use as an additional computational step when constructing clusters from networks.

## Experimental Procedures

### Datasets

We analyzed the robustness of several clustering algorithms, using both undirected graphs and raw proteomic data as inputs. First, we perturbed networks by randomly removing and adding edges. To provide a broad range of networks, we clustered five protein–protein interaction networks, a network of drug–drug side-effects, and a social network (supplemental Fig. S1):

(1) Comprehensive resource of mammalian complexes (CORUM): A literature-curated interactome (CORUM, https://mips.helmholtz-muenchen.de/corum/, downloaded September 2018) (8). Because CORUM is published as a list of protein complexes, not pairwise interactions, we first reduced the 2824 CORUM complexes among human proteins to their pairwise network. This produced a network of 39,563 protein–protein interactions (edges) between 3645 unique proteins (nodes). The original protein complexes were used as a ground-truth cluster set.
(2) BioGRID: A literature-curated interactome (https://downloads.thebiogrid.org/BioGRID file BIOGRID-ALL-3.5.186.tab3.txt, downloaded June 2020) (9). The full interactome of more nearly two million interactions was reduced to interactions between human proteins, producing a network with 571,848 interactions between 18,631 proteins.
(3) BioPlex: An interactome compiled from AP-MS experiments in HEK293T cells (https://bioplex.hms.harvard.edu file BioPlex_293T_Network_10K_Dec_2019.tsv, downloaded June 2020) (10). This network consists of 118,162 interactions between 13,689 proteins.
(4) Collins2007: An interactome merging two other high-confidence interactomes (12). Importantly, this network is weighted, with edge weights being equal to the published confidence score (minimum edge weight = 0.48, maximum weight = 0.99). As in (12), we analyzed the top 9074 protein pairs between 622 proteins.
(5) HuRI: An interactome of human proteins compiled from Y2H experiments (11). This network consists of 52,547 interactions between 7116 proteins.
(6) DrugBank: A drug–drug interaction network, which is a subset of the DrugBank database that lists drug pairs with known interactions, *i.e.*, drug pairs that have unwanted side-effects when taken together (DrugBank, https://snap.stanford.edu/biodata/datasets/10001/10001-ChCh-Miner.html, downloaded May 2019) (13). This network consists of 48,514 drug–drug interactions (edges) between 1514 unique drugs (nodes).
(7) email-Eu: A social network, consisting of anonymized emails between members of a European research institution (email-Eu, https://snap.stanford.edu/data/email-Eu-core.html, downloaded May 2019) (15, 20). Nodes in this network represent members of the institution and are connected by an edge if either person sent the other at least one email (16,063 edges between 1005 nodes). The original network was directed and contained self-interactions. For the purposes of this study, we removed self-interactions and modified it to be undirected. Each research institute member was a member of exactly one of 42 departments, and these department affiliations were used as a ground-truth cluster set.

All networks are undirected with no self-interactions. All networks are unweighted (*i.e.,* all edge weights = 1) except Collins2007 which is weighted by the published interaction confidence score (12). The CORUM and email-Eu networks had ground-truth cluster assignments.

Second, to explore the effect of injecting noise directly into the experimental data that provides the basis for network inference, we also clustered networks inferred from four sets of co-fractionation experiments collected by our laboratory (16, 17, 18, 19). These datasets were collected to reconstruct protein–protein interaction networks, using co-fractionation over size exchange chromatography as evidence of protein interaction. They were collected across different species and experimental conditions and represent a broad range of co-fractionation experiments: Kristensen *et al*. (18) studied the response of HeLa cells to epidermal growth factor over three biological replicates; Scott *et al*. (17) studied HeLa cell response to *Salmonella* infection, four replicates; Scott *et al*. (16) studied apoptotic Jerkat cells, three replicates; and Kerr *et al*. (19) studied HeLa cell response to interferon stimulation, four replicates. Each dataset is composed of thousands of co-fractionation profiles collected under two experimental conditions and repeated in at least three replicates. In this study, we treated each combination of replicate and experimental condition separately, of which there were 28 in total. To generate an interactome network from each replicate/condition, we used the PrInCE analysis pipeline with default settings (21). As in previous work (11, 22), our intent in so doing is not necessarily to argue that PrInCE represents the single most accurate approach for protein–protein interaction inference from co-fractionation data but rather that it is sufficiently representative of the kinds of machine-learning approaches used within the field (*e.g.,* (23, 24, 25)) that our conclusions will generalize more broadly. Three replicate/condition combinations produced interactomes with fewer than 50 pairwise interactions, likely because of poor data quality, and these were not analyzed in this study. Before adding noise, the remaining 25 datasets used for this study produced interactomes with between 143 and 14,358 interactions (6139 ± 3989, mean ± SD).

### Experimental Methodology

**Fig. 1:**
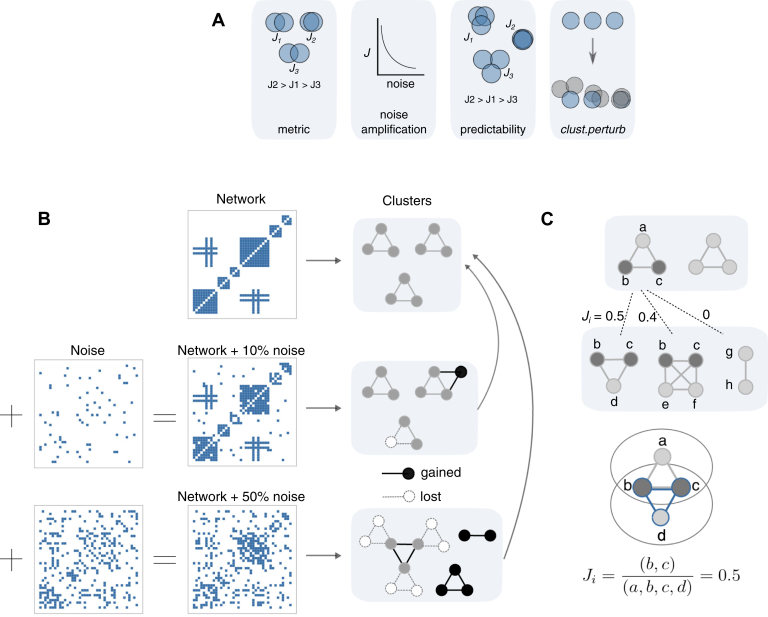
**Testing the effect of network noise on clustering.***A*, primary findings of this study. *B*, overview of methodological design. Networks with varying amounts of noise are clustered and results are compared to clusters derived from the original networks. *C*, cluster-wise comparisons are made using the Jaccard index.

To test the effects of network noise on clustering results, we added network-level noise by randomly rewiring network edges and clustered both noised and original networks using graph-based clustering algorithms. That is, a certain number of edges were removed from the network and replaced with the same number of edges not previously contained in the network. Thus, the size of the network was preserved while the fraction of rewired edges was varied. The fraction of rewired edges was calculated as a false positive rate, equal to the number of rewired edges divided by the total number of edges in the network. To avoid confusion with a network's inherent false positive rate, which comes from mistakes in the original edge list, we use the term “network noise level” to refer to the false positive rate of *in silico* changes made to the network. To track the effects of network noise on clustering, we compared clustering results from noised networks to the clustering results from the original network (Fig. 1*A*). We repeated this noised-to-original comparison for each clustering algorithm.

In addition to rewiring, we ran limited analyses with different levels of added or removed edges to examine whether our conclusions are specific to a particular type of noise (*i.e.,* false positives or false negatives). In these cases, a fraction of edges were removed, or edges not originally in the network were added, or both in differing amounts. Similar to rewiring, the number of added or removed edges was proportional to the size of the network. Noise levels analyzed were *fnoise* = 0%, 1%, 2%, 5%, 10%, 15%, 25%, 50%, and 100%. Because this produces 81 noise combinations, we also calculated the overall level of injected network noise as Δ*edges* = *f_add_* + *f_remove_*, *i.e.,* the sum of the added and removed fractions.

The effects of experimental noise on clustering results were measured in much the same way, except that noise was added to co-fractionation profiles before generating a network via PrInCE, rather than to the network directly. Noise was added to co-fractionation profiles by adding a normally distributed random number to the log-transformed value of each data point, with standard deviation equal to the noise level. That is, for each data point,

y′=y+fX,

where *y*′ is the noised log-transformed co-fractionation data, *y* is the original log-transformed co-fractionation data, *f* is the co-fractionation noise level, and *X* is a normally distributed random number with mean zero. Log-transformation was used because co-fractionation values tend to follow an approximately log-normal distribution. Although we employed a different method for adding noise to these co-fractionation datasets compared with the other 7 networks (adding noise to the co-fractionation profiles rather than the interactome generated from the profiles), importantly, the end result is the same: added or lost network edges before clustering.

### Choosing a Cluster-Wise and Set-Wise Similarity Metric

Many different metrics have been proposed to measure the similarity of two sets of clusters (2). These metrics implicitly trade off rewarding intracluster edges (true positives) and penalizing intercluster edges (false positives). Previous work has shown that this trade-off leads different cluster similarity metrics to measure distinct aspects of cluster similarity and to display nonintuitive behaviors (2). Therefore, we examined which set-wise similarity metrics, if any, adhered to intuitive notions of “cluster set similarity”, such as measuring complete similarity when sets are identical and less than complete similarity when they are not. Set-wise metrics assign a similarity value to entire sets of clustering results, *i.e.,* a single value for all clusters. This in contrast to cluster-wise metrics, which assign a similarity score to each cluster. Set-wise metrics are often computed as an average of cluster-wise metrics.

We analyzed whether set-wise metrics matched intuition in four scenarios:

(1) A similarity score of 1 is assigned to identical cluster sets and decreases as the number of nonidentical cluster assignments increases.
(2) The score is not biased by the number of clusters in either set.
(3) The score is not negatively affected by nodes participating in multiple clusters (“moonlighting”).
(4) The score penalizes situations where sets are nonidentical because of missing nodes in one set.

These were tested using simulated clustering sets of 1000 nodes assigned to 100 equal-sized clusters, before manipulation in each condition. Our analysis parallels that of Gates *et al.* (2). In this simulation, we tested six commonly used set-wise cluster similarity metrics:

• Normalized mutual information (26)
• Adjusted rand index (27)
• GA (3, 5, 7)
• Maximum matching ratio (3)
• F-measure
• Jaccard index

### Maximum Jaccard Index and Simple Counting Statistics

To measure whether cluster *i* in cluster set 1 is also contained in cluster set 2, we quantify cluster-wise similarity using the maximum Jaccard index (*J*), which is the number of nodes in common between two clusters divided by the total number of unique nodes in the two clusters (Fig. 1*B*). The cluster-wise Jaccard index *Ji* is calculated as

$$J_{i}\;=\;\max_{j}\left(\frac{overlap\left(cluster\;1_{i},\;cluster\;2_{j}\right)}{union\left(cluster\;1_{i},\;cluster\;2_{j}\right)}\right),$$

where *cluster1* is the noised cluster set, *cluster2* is the original cluster set, and *cluster1*_*i*_ and *cluster2*_*j*_ are single clusters from those sets. That is, the similarity score *Ji* of a cluster from a noised cluster set is equal to the maximum Jaccard index between that cluster and any cluster in the original set. Set-wise similarity *J* is quantified by averaging *Ji* over all clusters in the noised cluster set. That is,

$$J\;=\;avg\left(J_{i}\right)$$

In addition to metric *J*, we also employed simple counting statistics to measure the difference between cluster sets. These include the number of gained nodes (nodes present in a noised cluster that are not present in the best-match original cluster), the number of lost nodes (nodes not present in a noised cluster which are present in the best-match original cluster), and the number of rearranged cluster edges. The latter is defined as the absolute number edges different between a noised and original cluster set, *i.e.*, the number of edges one would need to add to or remove from the original cluster set to make it identical to the noise-added set.

### Clustering Algorithms

We analyzed the results of nine clustering algorithms: MCL (28), CO (java implementation at www.paccanarolab.com) (3), k-medoids (“k-medoids”, R function *pam*), walktrap clustering (“walktrap”, R function *walktrap.community*) (29), hierarchical clustering (“hierarchical”, R function *hclust*), MCODE (“mcode”, R function *mcode*) (34), Louvain clustering (“louvain”, R function *cluster_resolution*) (30), and Leiden clustering (“leiden”, R function *leiden*) (31). We also include a two-stage clustering algorithm of CO followed by MCL (“CO+MCL”) for certain analyses. We chose these algorithms to provide a broad range of algorithms, including those popular for clustering interactomes (CO, MCL, CO+MCL, MCODE) and biological networks (Louvain, Leiden) as well as more general clustering algorithms (hierarchical clustering, k-medoids, Walktrap). CO+MCL has been used specifically to avoid large clusters sometimes predicted by CO (24, 32). The k-medoids algorithm is similar to k-means, although we used the less-common k-medoids because its R implementation (*pam*) allows analysis to start from an adjacency matrix, whereas common R implementations of k-means do not. This is important because our method adds noise to adjacency matrices (Fig. 1*A*).

Parameters for all clustering algorithms were chosen by grid search optimization. Parameter optimization was performed with the original networks. Because CORUM and email-Eu had ground truth cluster assignments, we chose the parameter sets that maximized set-wise *J* between clusters derived from the original network and the ground truth clusters. For other networks, we chose the parameter set that maximized the silhouette score of the cluster assignments, which selects well-separated clusters whose members are tightly connected by pairwise edges. Parameter ranges and optimized values are given in Table 1. Parameters are:

• P (CO, CO+MCL): Penalty term modeling the number of unknown edges in a network.
• Dens (CO, CO+MCL): Minimum cluster density (fraction of filled edges between cluster members).
• I (CO+MCL, MCL): Expansion parameter.
• Nclusters (k-Medoids, hierarchical): Explicitly controls number of clusters.
• Steps (walktrap): Number of random walks.
• Resolution (louvain, leiden): Controls the size of clusters.
• Fluff (mcode): Boolean, expand cluster cores by one shell outward?
• Haircut (mcode): Boolean, remove singly connected nodes from clusters?
• vwp (mcode): Vertex weight percentage, controls the size of clusters.
• fdt (mcode): Cluster density cutoff.

**Table 1:** Parameter choices and ranges for clustering algorithms

Clustering algorithm	Optimal parameter choices (CORUM)	Parameter ranges	Nonoptimal parameter choices
CO	P = 500	P: [1, 50, 100, 500, 5000]	P = 2
	Dens = 0.1	Dens: [0, 0.1, 0.2, 0.3, 0.4]	Dens = 0.3
CO + MCL	P = 500	P: [1, 50, 100, 500, 5000]	P = 2
	Dens = 0.1	Dens: [0, 0.1, 0.2, 0.3, 0.4]	Dens = 0.3
	I = 4	I: [1, 2, 4, 8, 15, 20, 50]	I = 2
MCL	I = 2	I: [1, 2, 4, 8, 15, 20, 50]	I = 4
k-Med	Nclusters = 1500	Nclusters: [50, 100, 250, 500, 1000, 1500, 2000, 5000]	Nclusters = 1000
walktrap	Steps = 2	Steps: [2, 4, 6, 8, 10, 12]	Steps = 10
Hierarchical	Nclusters = 1500	Nclusters: [250, 500, 1000, 1500, 2000, 5000]	Nclusters = 1000
MCODE	Fluff = False	Fluff: [True, False]	Fluff = False
	Haircut = True	Haircut: [True, False]	Haircut = False
	vwp = 0.1	vwp: [0, 0.25, 0.5, 0.75, 1]	vwp = 0
	fdt = 0.5	fdt: [0, 0.25, 0.5, 0.75, 1]	fdt = 1
Louvain	Resolution = 15	Resolution: [0, 0.25, 0.5, 0.75, 1, 1.5, 2, 4, 8, 10, 15, 20, 100]	Resolution = 2
Leiden	Resolution = 2	Resolution: [0, 0.25, 0.5, 0.75, 1, 1.5, 2, 4, 8, 10, 15, 20, 100]	Resolution = 1

CO, ClusterONE; CORUM, comprehensive resource of mammalian complexes; MCL, Markov clustering.

For full parameter explanations see (3) (CO) (33), (k-Medoids) (31), (Leiden) (30), (Louvain) (34), (MCODE) (28), (MCL), and (29) (walktrap).

### *clust.perturb*: A Tool for Assessing Cluster Reliability

*clust.perturb* is both an open source R package (https://github.com/fosterlab/clust-perturb) and web application (https://rstacey.shinyapps.io/clust-perturb-tool/, R shiny) designed to assess cluster reproducibility by detecting the tendency for clusters to change after random perturbations are applied to the clustered network. It is designed as a general-purpose wrapper to clustering algorithms, which will return both the original clusters and measures of the reproducibility with which individual clusters and proteins are detected by the algorithm. The R package can be used to assess clusters from any clustering algorithm, whereas the web application uses three default clustering algorithms (hierarchical clustering, MCL, and k-Medoids). Networks are perturbed by rewiring edges as described in this paper, and the networks are input as edge lists. *Clust.perturb* takes three input parameters and returns two outputs. The input parameters are clustering algorithm; number of iterations; and noise level, quantified as the fraction of network edges that are rewired. The outputs are *repJ*, a measure of cluster reliability, which is equal to the cluster's average *Ji* over all noise iterations and *fnode*, a measure of node reproducibility within a cluster, which is equal to the frequency with which that node occurs in best-matched clusters divided by the number of noise iterations.

## Results

In this study, we investigated the degree to which graph-based clustering of biological and social networks are contaminated by network-level noise. To do so, we sought to address four questions (Fig. 1*A*). First, we established a suitable metric to measure changes in clustering solutions after injection of noise into a network. Second, we used this metric to demonstrate that clustering amplifies network noise, *i.e.,* the ratio of network level noise to set-wise Jaccard index *J* is greater than 1, such that injection of a small degree of noise into a network can result in dramatic changes to its clustering. Third, we demonstrated that it is possible to predict which clusters will be most affected by noise. Finally, we developed a tool (*clust.perturb*) that estimates the reproducibility of cluster assignments.

### An Intuitive Metric for Measuring Clustering Similarity

**Fig. 2:**
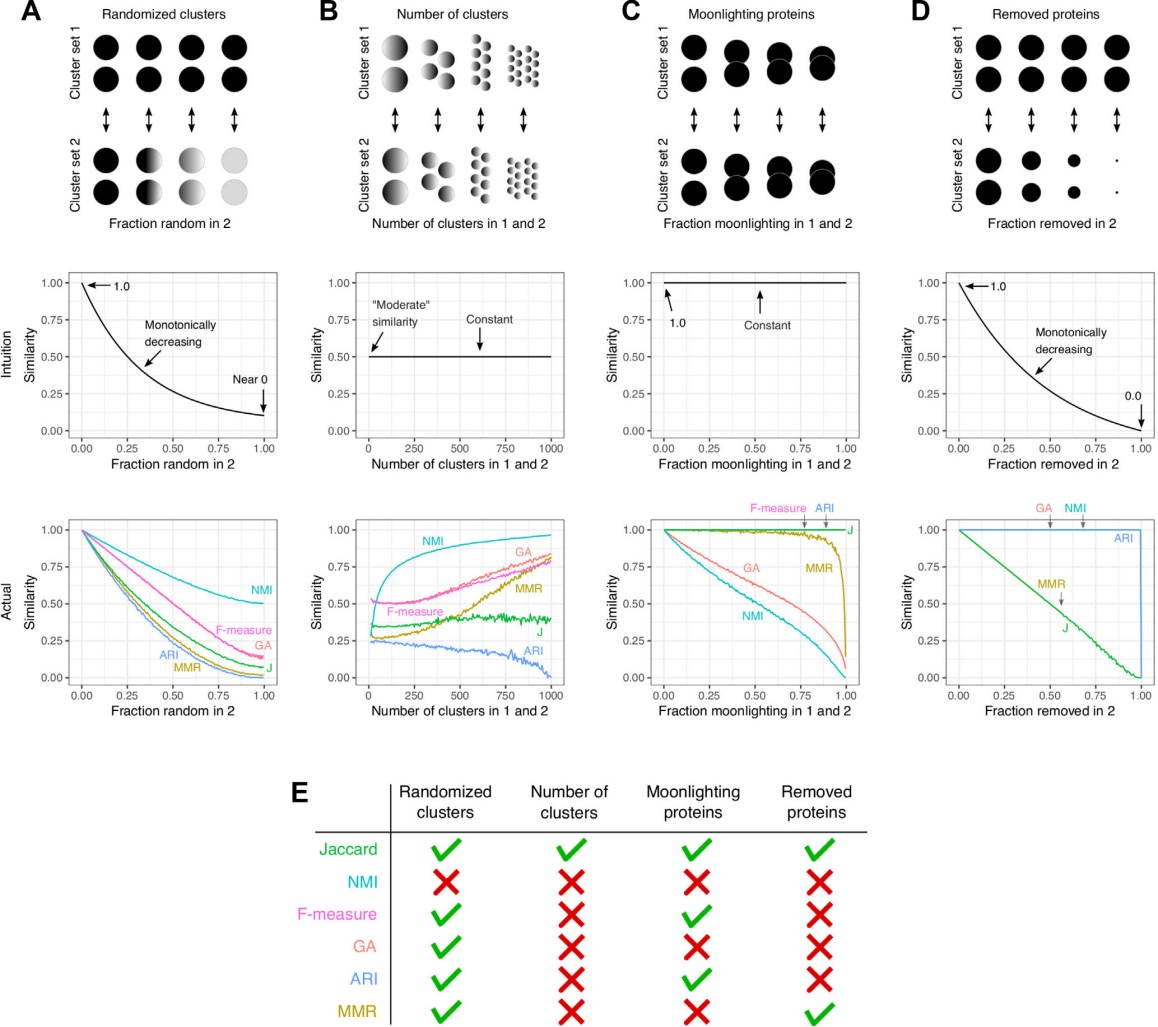
**Maximum Jaccard index (*J*) is consistent with intuitive notions of “cluster set similarity” in all four cases.** In all cases, 1000 nodes are assigned to clusters, except D where the number of nodes in set 2 is varied. *A*, set 1: 1000 nodes assigned to 100 clusters of equal size. Set 2: identical to set 1, aside from a variable fraction of nodes randomly assigned to an existing cluster. *B*, in all comparisons, 50% of cluster assignments are identical, and the number of clusters is varied. Set 1: 500 nodes assigned to a variable number of clusters of equal size, with the remaining 500 nodes randomly assigned to an existing cluster. Set 2: identical to set 1 for the first 500 nodes, but different random assignments for the remaining 500 nodes. *C*, cluster sets 1 and 2 are identical in all comparisons, and the number of nodes assigned to multiple clusters is varied to simulate “moonlighting” nodes. Sets 1 and 2: 1000 potentially nonunique nodes are assigned to 100 clusters of equal size. At fraction = 0, all nodes are unique, whereas at fraction = 1, all nodes are the same node. *D*, set 1: 1000 nodes assigned to 100 clusters of equal size. Set 2 is identical to Set 1 before a fraction of Set 2 nodes are removed. At fraction = 0, both sets are identical, whereas at fraction = 1, set 2 is empty. ARI, adjusted rand index; GA, geometric accuracy; MMR, maximum matching ratio; NMI, normalized mutual information.

Measuring the effects of noise on clustering results requires a metric for quantifying the difference between two cluster sets. Because quantifying clustering similarity requires choosing how to reward true positives (within-cluster edges) and penalize false negatives (between-cluster edges), this task can be surprisingly ambiguous. There exist many commonly used cluster similarity metrics (35) with biases that result in unintuitive behavior (2). Therefore, we tested a number of commonly used metrics with the goal of ensuring that we are indeed measuring an intuitive notion of “cluster set similarity”. Confirming previous results (2), we found that most metrics failed to adhere to intuition (Fig. 2). For example, geometric accuracy (GA) and normalized mutual information can both measure disagreement between completely identical sets (in cases of “moonlighting”, *i.e.,* where nodes are assigned to multiple clusters, Fig. 2*C*) and measure perfect agreement between nonidentical sets (when a cluster set contains nodes not contained in the other set, Fig. 2*D*). However, the maximum Jaccard index *J* (Fig. 1*B*) behaved intuitively in all situations tested. This metric also has the benefit of measuring both cluster-wise and set-wise similarities (*J* and *Ji,* respectively). We therefore use *J* and *Ji* throughout this study.

### Clustering Amplifies Network-Level Noise

**Fig. 3:**
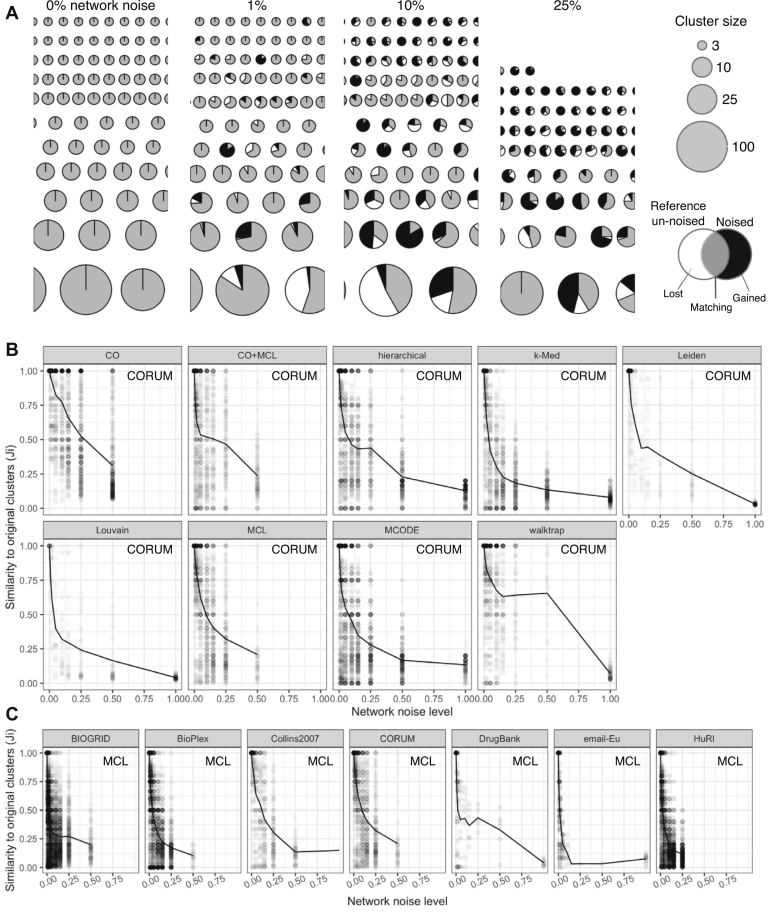
**Clustering amplifies network noise.***A*, subset of the cluster sets produced by clustering the CORUM network with 0 to 25% network noise. Color shows the fraction of proteins overlapping with original clusters, proteins lost after adding noise, and proteins gained. Clusters were identified with the MCL algorithm. *B*, quantifying the effects of CORUM network noise using set-wise metric *J* (*line*) and cluster-wise metric *Ji* (*scatter*) *Line* shows average *Ji* (*J*). *C*, quantifying the effects of network noise on all seven networks. Algorithm MCL. CO, ClusterONE; CORUM, comprehensive resource of mammalian complexes; MCL, Markov clustering.

Because graph-based clusters are generated from a network, one would expect that changes to the network would also lead to changes in clusters. Indeed, this is the case: as network-level errors increase in the CORUM network, cluster sets both lose and gain proteins when compared with sets of clusters derived from the error-free network (Fig. 3*A*). This response to injected network noise was quantified by our chosen cluster-wise and set-wise metrics (*Ji* and *J*, respectively) (Fig. 3*B*). The injected network errors in this case are random connections between proteins that do not necessarily share any biological role and the removal of true positives, which should be accompanied by a loss of biological plausibility in the clustered proteins. Therefore, as a control analysis, we confirmed that clusters derived from noised networks are less enriched for Gene Ontology (GO) terms than clusters derived from the CORUM network without added noise (supplemental Fig. S2).

While it is to be expected that large amounts of injected network noise lead to changes in clustering results, we find that this is the case for even small amounts of injected network noise. For all clustering algorithms, an alteration of 1% of the binary interactions (edges) in the CORUM network resulted in the alteration of at least 13% of clusters in a set (ClusterONE [CO]) and at most 84% of clusters (Louvain). Measuring the set-wise change, an introduction of 1% network noise lead to a 4 to 27% change in clustering results as quantified by *J* (*J* = 0.96 to *J* = 0.73), and a 2% noise resulted in set-wise clustering differences between 8 and 41% (Fig. 3*B*). Additionally, there was a positive relationship between cluster size and reproducibility, as shown by multiple linear regression between *Ji* (dependent variable) and network noise level, algorithm, and cluster size (beta = 0.2 × 10^−4^, *p* = 1.3 × 10^−6^; model R^2^ = 0.42). That is, small clusters were significantly less reproducible. Importantly, the variability of cluster sets measured here is because of network noise and not inherent randomness in the clustering algorithms: all of the algorithms analyzed here are deterministic, meaning the clustering does not vary if the network is unchanged (see Fig. 3*B*, *Ji* = 1 when noise = 0).

Because CORUM represents a highly curated, “ground truth” set of human protein complexes, we also tested the effects of noise injection in noisier human interactomes inferred from high-throughput studies. Amplification of network noise by clustering was also seen when clustering AP-MS and Y2H interaction networks in addition to CORUM, as well as other biological and nonbiological networks (Fig. 3*C*; see supplemental Fig. S3 for full network-*versus*-algorithm analysis). As was observed for CORUM, small network changes (rewiring 1–2% of the edges) lead to substantial changes in clustering results, including total loss of some clusters. However, in general, the degree of noise sensitivity was greater for the high-throughput networks: 1% and 2% network noise lead to average values of *J* = 0.58 and *J* = 0.50 for non-CORUM networks, compared with *J* = 0.76 and *J* = 0.68 for CORUM, respectively. (This difference could be because of CORUM interactome being constructed from already well-separated complexes, making the network more robust to clustering.) The maximum cluster rearrangement at 1% network noise was *J* = 0.17 for BioGRID and hierarchical clustering. Therefore, we conclude that amplification of network noise is a general property of graph-based clustering, as applied to many different types of networks.

Clustering appears to be sensitive to network noise when measured by *J*. However, set-wise metrics can be misleading (Fig. 2), so we also sought to confirm that substantial clustering rearrangements were occurring through visual inspection and simple summary statistics. Supplemental Figure S4, *A*–*E* shows visually the results of clustering the binarized CORUM network via Markov clustering (MCL) and the extent of gained/lost proteins at various noise levels. Consistent with conclusions based on metric *J*, after 1% of the binary interactions were rewired, more than a third of clusters underwent some rearrangement (102/305 clusters), with clusters losing 0.88 proteins and gaining 1.82 proteins on average (supplemental Fig. S4*F*). We also counted the number of lost and gained cluster edges, *i.e.,* the number of edges one would need to alter in the original cluster set to arrive at a noised cluster set (supplemental Fig. S4*F*). This permitted us to directly compare the number of rewired network edges to the number of rearranged cluster edges. We saw that at low levels of network noise (1% network noise level), a single rewired network edge produced 38.2 rearranged cluster edges on average, consistent with clustering amplifying network noise.

Network noise commonly involves both the absence of truly occurring edges (false negatives) and the presence of spurious edges (false positives). In the rewiring experiments above, we simulated the addition of both false positives and false negatives simultaneously. We also asked whether the consequences of network noise for clustering robustness varied when false positives and false negatives were added in varying proportions. We saw that regardless of whether edges were removed, added, or rewired, clustering consistently amplified low levels of injected network noise (supplemental Fig. S5*B*, red *versus* blue). However, at higher noise levels, we found that for most clustering algorithms edge removal generally had a greater effect than edge addition.

Finally, we considered the possibility that the sensitivity to network noise that we observed could be specific to the set of clustering algorithm parameters that yielded the optimal clustering solution. Although it is unlikely that this result is unique to a parameter set, given that we employed multiple algorithms and two optimization schemes (optimal ground truth similarity and optimal silhouette score), we wanted to test whether noise sensitivity persisted when clustering the same network with the same algorithm but specifically selecting a different, nonoptimal parameter set (Table 1). Clustering CORUM with nonoptimal parameters, 1% network noise lead to a minimal rearrangement of *J* = 0.85 (CO) and a maximal rearrangement of *J* = 0.55 (Louvain), compared with values *J* = 0.95 and *J* = 0.73 for cluster sets using optimized parameters (Fig. 3). Averaged over all algorithms, optimal parameters produced *J* = 0.83, and nonoptimal parameters produced *J* = 0.72, meaning optimal parameters also yielded more robust clustering on average. Therefore, sensitivity to network noise appeared to be a general feature of the clustering algorithms studied here, rather than a consequence of parameter optimization.

### Clustering Results Derived From Experimental Datasets Are Also Poorly Reproducible

**Fig. 4:**
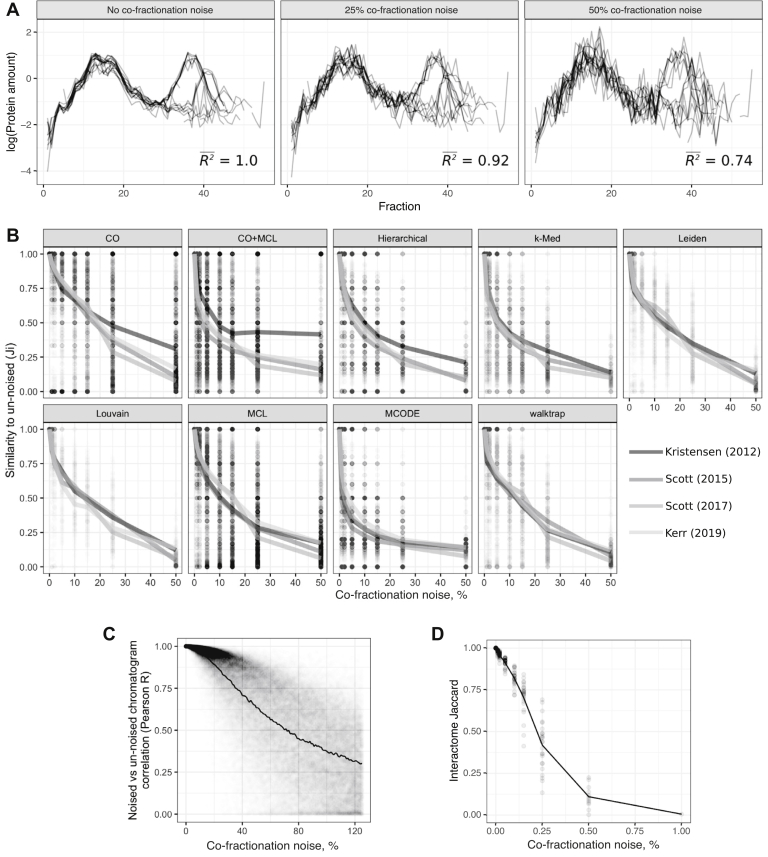
**Experimental noise also affects clustering results.***A*, co-fractionation profiles of 26S proteasomal proteins with no added noise (left), 25% noise (middle), and 50% noise (right). Average R^2^ values shown are calculated between each co-fractionation profile before and after adding noise. In the “No noise” case, these are the same profiles, hence perfect correlation. *B*, effects of adding co-fractionation noise on clustering results, measured with *J* (lines) and *Ji* (points). *C*, quantifying the degree to which added co-fractionation noise degrades co-fractionation profiles, as measured by Pearson correlation between noised and original co-fractionation profiles. *D*, effect of co-fractionation noise on interactomes, as measured by Jaccard index between noised and original interactome. Each dot is a dataset, and the line shows the average. CO, ClusterONE; MCL, Markov clustering.

Having established that graph-based clustering techniques amplify noise injected into binary interaction networks, we also asked whether these algorithms would also amplify noise at the level of the experimental data that enables network inference. To address this possibility, we analyzed the impact of noise injection on network inference from co-fractionation data generated within our laboratory (Methods). In this analysis, we added noise directly to the underlying proteomic data rather than the network (Fig. 4*A*), then subsequently performed network inference on the noisy proteomic data, to investigate the impact of experimental noise on clustering. Consistent with previous results from the binarized CORUM network, injecting relatively insignificant levels of noise into experimental co-fractionation datasets resulted in substantially altered clustering results (Fig. 4*B*). For example, although co-fractionation profiles with 1% added noise were highly similar to profiles without added noise (average Pearson R^2^ = 0.9996, Fig. 4*D*), clustering these two noise levels could produce clustering sets with large differences (Fig. 4*B*, *e.g.,* CO + MCL *J* = 0.68, MCODE *J* = 0.57), the magnitude of which were comparable to or larger than those observed in CORUM. Importantly, injecting small amounts of noise also had small effects on the predicted interactome network, as interactomes derived from co-fractionation profiles with 1% added noise were largely similar to interactomes derived from profiles with no noise (Jaccard = 0.96, Fig. 4*D*) and similarly for 2% added noise (Jaccard = 0.93). That is, slight alterations to experimental data could abolish a large proportion of clusters, while having a lesser impact on the interactomes from which the clusters are derived. This is consistent with the results obtained from analysis of CORUM and reflects the amplification of both network-level noise, as well as noise in the underlying experimental data, by graph-based clustering.

### The Response of Individual Clusters to Network Noise Is Reproducible

Our results thus far indicate that the clustering algorithms studied here are globally sensitive to small levels of noise in the underlying networks. However, not all clusters are equally affected by this sensitivity to noise. For example, adding 5% noise to the CORUM network and clustering with k-Medoids produced a cluster set with moderate similarity to the original set (*J* = 0.42 at 5% noise, Fig. 3*B*, k-Med panel). However, within that set, some clusters remained unchanged (*Ji* = 1, top), whereas others were entirely removed (*Ji* = 0, bottom). We investigated the consistency of this pattern, that is, whether some clusters tended to be more stable in response to network noise than others. We reasoned that, if some clusters are consistently reproducible in response to simulated network noise, then it may be possible to identify clusters that will be robust to future, real-world alterations of the network, *e.g.,* the collection of data in future experiments.

**Fig. 5:**
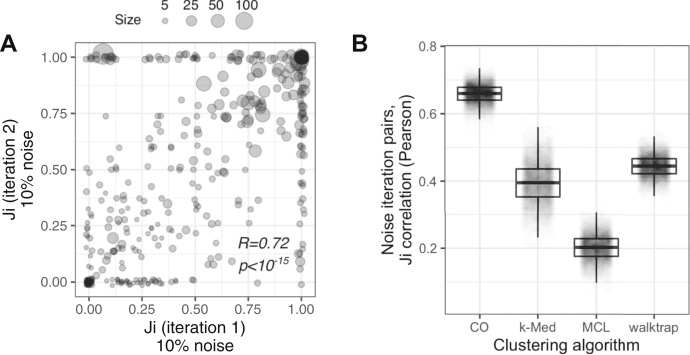
**Response of individual clusters to network noise is consistent across noise iterations.***A*, *Ji* values between clusters of the CORUM network (ClusterONE) and two noise iterations (ClusterONE plus 10% shuffling). Pearson correlation. *B*, Pearson correlation values between pairs of noise iterations for four clustering algorithms. CO, ClusterONE; CORUM, comprehensive resource of mammalian complexes; MCL, Markov clustering.

To quantify cluster stability, we performed multiple iterations of noise injection and clustering using the CORUM network and then calculated *Ji* between the original cluster set and each iteration. That is, for each of the original clusters, we calculated *N* values of *Ji*, where *N* is the number of noise iterations. If some clusters are consistently stable (or unstable) in response to noise, the *Ji* values should be consistently high (or low) across noise iterations. This is indeed what we observe. Measuring this consistency as a correlation in the Jaccard index between iterations, Figure 5*A* shows *Ji* values for two iterations of clustering, using the CO algorithm, across independent noise injections. These iterations are significantly correlated (R = 0.72, *p* < 10^−15^, Pearson correlation; CO). For all clustering algorithms studied here, the reproducibility of individual clusters was correlated between random noise iterations, with CO having the highest correlation and MCL the lowest (Fig. 5*B*). Across algorithms, a cluster's reproducibility correlates with its density, *i.e.,* fraction of intracluster edges (Spearman R = 0.35, *p* < 10^−16^). Taken together, this suggests that a cluster's tendency to “break” in response to injection of network noise is predictable and that more reproducible clusters are more supported by intracluster edges in the underlying network.

### A Software Tool for Predicting Cluster Reproducibility (*clust.perturb*)

**Fig. 6:**
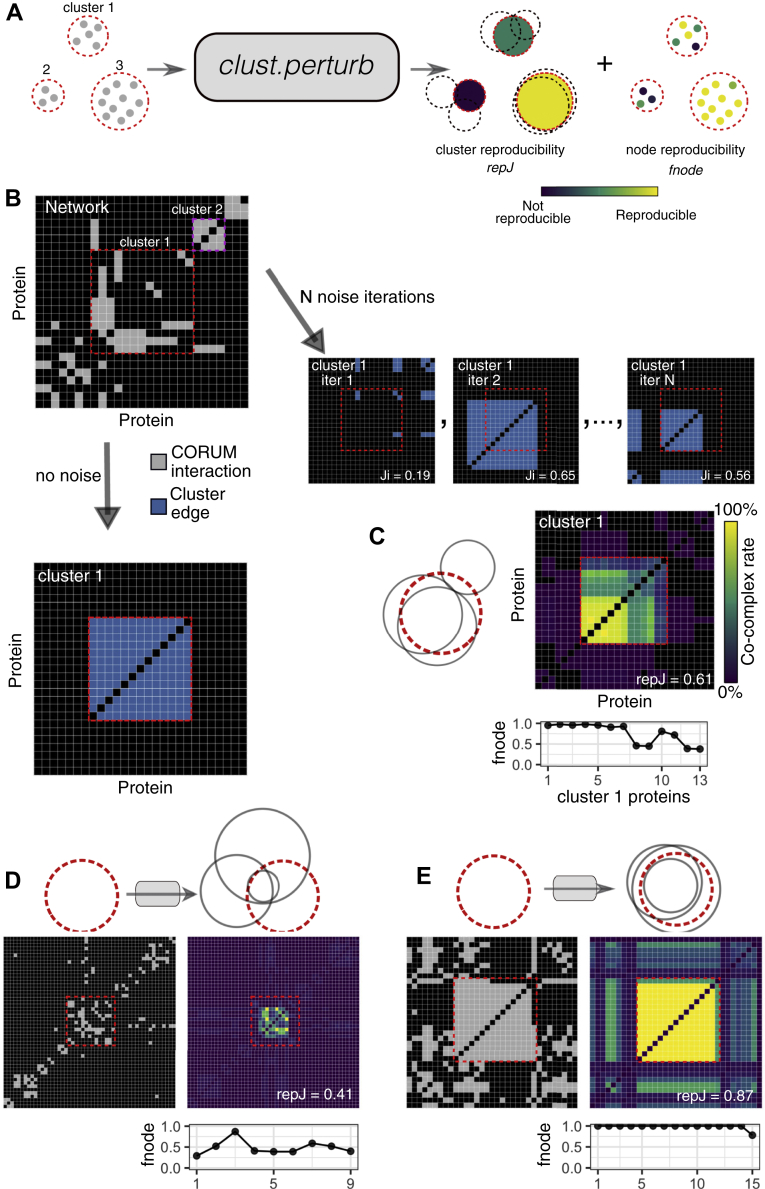
***clust.perturb* measures cluster and node reproducibility.***A*, schematic *clust.perturb* clusters a network with and without noise. A cluster's reproducibility is its average overlap with best-match clusters across noise iterations (*repJ*). Node reproducibility is the frequency with which a cluster's nodes appear in best-match noise clusters, divided by the number of noise iterations (*fnode*). *B*, network adjacency matrix showing a subset of the CORUM network (*gray*) and a 13-protein cluster identified in the network (cluster 1, *blue*). Adjacency matrices for best-match clusters from 3 noise iterations are also shown (10% noise). *C*, average adjacency matrix for cluster 1 across 100 noise iterations. *fnode* values for each protein in cluster 1 are shown. Venn diagram shows overlap between cluster 1 (*red*) and best-match clusters from three noise iterations (*gray*). *D* cluster with low reproducibility. *E* cluster with high reproducibility. ClusterONE, 10% network noise. CORUM, comprehensive resource of mammalian complexes.

Some clusters are consistently robust when the network is randomly altered *in silico*. If the simulated network noise is representative of real-world network alterations, it should be possible to predict the effect of future alterations to the network and thereby identify robust clusters. To this end, we developed *clust.perturb*, an R-based tool for measuring cluster reproducibility by randomly perturbing the network. *clust.perturb* takes a network as input and returns two scores, *repJ* and *fnode*, which quantify the reproducibility of clusters and the reproducibility nodes within clusters, respectively (Fig. 6*A*). Following the previous analysis, *clust.perturb* first clusters the network, then performs *N* iterations of clustering with network noise, yielding *N* values of *Ji* for each cluster from the original cluster set. *repJ* is then calculated as the average of these *Ji* values. For example, CO identifies a 13-protein cluster in the CORUM network loosely corresponding to the G alpha-13-Hax-1-cortactin-Rac complex (Fig. 6*B*, left). Over multiple network noise iterations (Fig. 6*B*), seven proteins in the original complex tend to remain co-clustered (Fig. 6*C*, top yellow), whereas the other six proteins do so less consistently. On aggregate, this cluster is partially reproduced, reflected by its reproducibility score of *repJ* = 0.61 (100 iterations). Other clusters are effectively “lost” when the network is altered (Fig. 6*D*, *repJ* = 0.41), whereas others still remain largely unchanged (Fig. 6*E*, *repJ* = 0.87).

In addition to scoring the reproducibility of each cluster, *clust.perturb* also scores the reproducibility with which each node (*e.g.,* protein) within a cluster is associated with that cluster, by counting the frequency with which it is re-clustered across noise iterations. A score is assigned to each node (*fnode*) based on the frequency with which it occurs in the closest matching noised cluster. For example in Figure 6*B*, *fnode* values close to 1 reflect the fact that these proteins are present in the best-matching cluster in nearly all noise iterations, whereas lower *fnode* values identify proteins that “drop out” of clusters.

*clust.perturb* requires two parameters in addition to the network and choice of clustering algorithm: number of noise iterations (*N*) and magnitude of network noise (*m*). We sought to establish sensible defaults for these parameters. Because the running time scales linearly with *N*, *clust.perturb* will be time-intensive if the original clustering algorithm is time-intensive. To find the minimum iterations necessary to adequately estimate a cluster's reproducibility, we took *N* = 100 iterations as a “final” value, and we calculated how quickly *repJ* converged to that value over successive iterations. After a single iteration, the average absolute error for a given *repJ* was 0.16, and after 25 iterations, it was 0.03 (CO, 10% network noise) (supplemental Fig. S6*A*). For all algorithms, a single iteration was sufficient to estimate the final *repJ* with a median error of 0.15 (supplemental Fig. S6*B*). Thus, we find that *repJ* converges relatively quickly, and a few iterations are often adequate to accurately approximate cluster robustness. Next, the noise magnitude *m* should be chosen to properly resolve *repJ* values: if a noise level is too small, most clusters will be unchanged and the resolving power will be poor (left, Fig. 6*C*), whereas the opposite is true if noise is too great (right). In general, we found a noise level of 10% was sufficient to resolve clusters generated by all algorithms (Fig. 6*C* center).

### Validating *clust.perturb* Using Biological Evidence

**Fig. 7:**
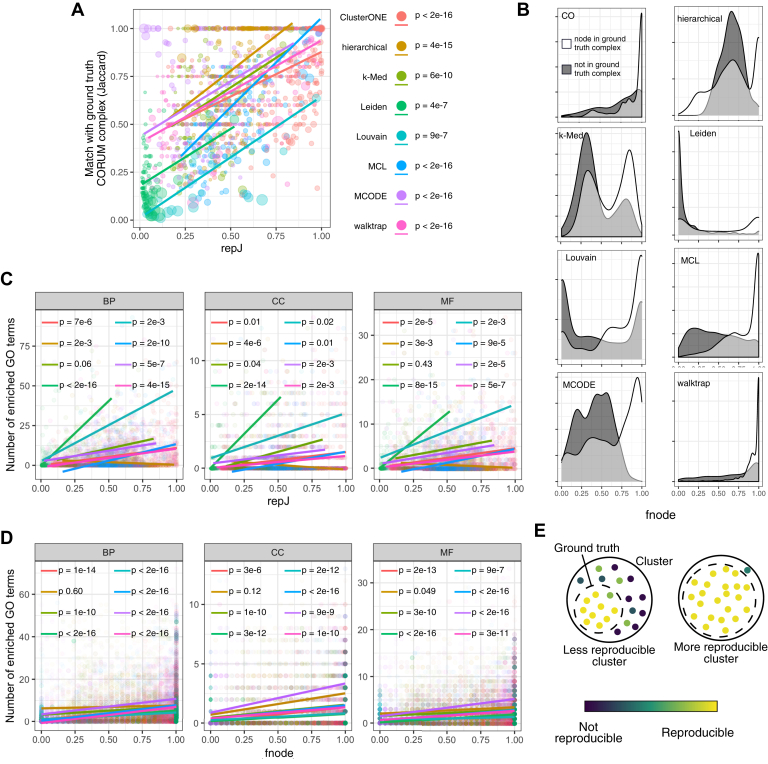
**Reproducible clusters and nodes measured by *clust.perturb* are associated with ground truth communities.***A*, clusters with high *repJ* values are more closely associated with a CORUM complex, as measured by Jaccard index. The *p*-values are for the *repJ* term in the linear regression model, where *CORUM_jaccard* (dependent) was predicted by *repJ* and *cluster_size* (independent variables). *B*, density plots of *fnode* values for nodes in the ground truth CORUM complex (*white*) and nodes not in ground truth (*gray*). *C*, number of enriched GO terms for a cluster correlates with its *repJ* value. The *p*-values for *repJ* term linear regression model, where *N_enriched_GO* (dependent) was predicted by *repJ* and *cluster_size* (independent variables). *D*, proteins with high *fnode* tend to share GO terms that their assigned cluster is enriched for. *E*, nodes “lost” in noise iterations are less likely to be from the ground truth complex, and similarly clusters more tightly associated with a ground truth complex are more reproducible. CO, ClusterONE; GO, Gene Ontology; MCL, Markov clustering. BP, biological process; CC, cellular compartment; CORUM, comprehensive resource of mammalian complexes; MF, molecular function.

We next sought to validate the reproducibility measures, *repJ* and *fnode*, calculated by *clust.perturb*. Specifically, do reproducible clusters and nodes correspond to meaningful features of the network? To answer this, we used the original CORUM complexes, which form the ground truth clusters for the network and GO terms. First, we investigated whether reproducible clusters more closely correspond to ground truth CORUM complexes. To do so, we matched each original cluster to its closest CORUM complex using the maximum Jaccard value. Indeed, for all clustering algorithms, we found a significant positive correlation between *repJ* and association with a CORUM complex (Fig. 7*A*). Similarly, cluster nodes with lower *fnode* scores tended to be proteins outside of the best-match ground truth CORUM complex (Fig. 7*B*). This association between reproducibility and ground truth persists even when clustering “noisier” interactomes such as BioGRID: following the analysis in Figure 7*A*, *repJ* scores for BioGRID clusters are significantly correlated with the degree to which the clusters match a CORUM complex, as quantified by Jaccard (R = 0.21, *p* < 2^−16^). That is, there was a strong, significant association between reproducibility and ground truth for both *repJ* and *fnode* measures.

We next investigated the association between reproducibility and GO enrichment (hypergeometric test with Benjamini–Hochberg correction, q < 0.05; GO terms filtered to >5 and <100 annotations among unique CORUM proteins). For each cluster from the original CORUM network, we counted the number of enriched GO terms in each GO ontology. For all algorithms studied except k-Med, possibly because of few clusters and therefore small sample size, the number of enriched GO terms was significantly positively associated with a cluster's *repJ* score, even when controlling for the size of cluster (multiple linear regression, *nGO* dependent variable, *repJ* and *cluster_size* independent; Fig. 7*C*). Correspondingly, across all algorithms, 92% of complexes with *repJ* > 0.8 were enriched for at least one GO term, compared with 47% of complexes with *repJ* < 0.5. We next investigated whether a protein's *fnode* score was associated with GO enrichment by analyzing whether proteins assigned to a cluster shared one of that cluster's enriched GO terms. Indeed, for all ontologies and algorithms except hierarchical clustering, we found a strong, significant pattern for more reproducible nodes to share the cluster's enriched GO terms (Fig. 7*D*). Taken together, these results suggest that reproducible clusters, as measured by *clust.perturb*, more closely align to the network's ground truth and that cluster nodes with poor reproducibility are more likely to be spuriously assigned to the cluster (Fig. 7*E*).

### *clust.perturb* Predicts Reproducibility Across Real-World Network Alterations

**Fig. 8:**
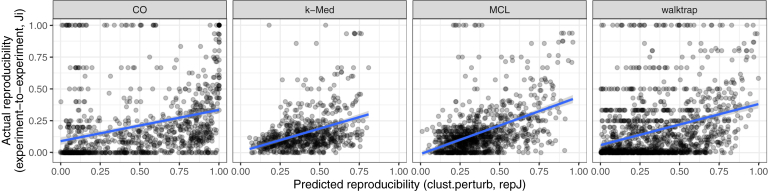
***clust.perturb* predicts real-world, experiment-to-experiment cluster reproducibility.** Two cluster sets were constructed from two independent networks from the same experiment (replicates from the same experiment were randomly separated into two groups), and *Ji* was calculated between the sets, simulating real-world network variation (*x*-axis). The predicted reproducibility *repJ* was calculated for each cluster by *clust.perturb* (*y*-axis). Each *dot* represents a cluster. *Blue lines* show best linear fit. CO, ClusterONE; MCL, Markov clustering.

If clusters have a consistent response to random, simulated network noise (Fig. 5), then it should be possible to predict which clusters will be robust to future, real-world network alterations. We tested whether this is indeed the case. We constructed two independent networks from each of the eight co-fractionation datasets by randomly separating each datasets' replicates into two groups and taking each network as the union of unique edges from each group. After clustering each pair of networks, it was possible to calculate predicted reproducibility (*repJ*) and actual reproducibility between experimental replicates (*Ji*) for each cluster. Figure 8 shows that these values were correlated for all clustering algorithms. Thus, we find that *clust.perturb* can accurately predict which clusters will fail to be reproduced in a second experiment (Fig. 8). On the basis of this observation, we suggest that *clust.perturb* may be particularly useful for planning follow-up studies in situations where failure to reproduce clusters that were apparent in an initial experiment would be costly or time-consuming.

## Discussion

We find that graph-based clustering amplifies network noise. Indeed, this phenomenon is repeatedly observed across nine different graph-based clustering algorithms and seven different networks. Consequently, in the presence of very minor random network-to-network variations (*i.e.,* noise), clustering results tend to be poorly reproducible. Even small changes to a network, such as rewiring 1% of edges, lead to cluster results rearranging by more than 25%. This phenomenon represents more than network errors simply being propagated to the level of clusters. Instead, network alterations appear magnified by clustering, such that the number of altered cluster edges can be many times the number of altered network edges. Importantly, this variance in cluster sets is because of network noise and not inherent randomness in the clustering algorithms: for all algorithms here except Leiden clustering, cluster sets do not vary if the network is unchanged (for Leiden, we set the random number generator seed to achieve deterministic behavior).

Studies that attempt to experimentally validate these clusters or otherwise include them in downstream analyses are vulnerable to this property and may ultimately analyze spurious arrangements particular to one dataset, rather than clusters with general, real-world meaning. This point is important because networks, especially biological networks such as protein–protein interactomes, often contain errors in the range studied here (*e.g.,* 10–50% false positives). If clustering accentuates these errors, then graph-based network clustering as a tool may be less useful to researchers than expected, at least in some contexts. This said, more work remains to be done to explain the different performance of these diverse clustering algorithms. For example, we did not fully explore how reproducibility is affected by algorithm-specific parameter choices. It is possible that some algorithms have parameter ranges that produce more stable clusters than shown here; although in general, we found that parameters that maximized performance also maximized reproducibility.

One explanation for this noise sensitivity is that graph-based clustering is an inherently ill-posed problem (36, 37). That is, there are many sets of clusters that could be described by a given network. For example, a fully connected network of three nodes and three edges could be a result of a three-member cluster, three two-member clusters, or any combination of them. Larger networks have even greater numbers of potential clusterings, especially in situations where subsets of clusters exist, such as the 40S and 60S subunits of the full 80S ribosome. This means that the solution space of clustering contains many solutions that are approximately equally correct, and without more constraints, it may be easy for noise to result in the selection of one over the others.

Our analysis also recapitulates previous observations that measuring the similarity of two sets of clusters can be ambiguous (2). Previous studies have looked at the robustness of clustering results to noise, particularly interactome clustering (4, 5, 6, 7). Notably, Broheé *et al*. (5) reported that clustering results appeared robust to both the addition and removal of interactions from the interactome. However, because of the ambiguity and difficulty with measuring the similarity between clustering sets, it is possible that these studies were, in fact, measuring something other than the “similarity” that we propose would correspond to a biologist's intuition about how clusters should behave. Indeed, Broheé *et al*. (5) use GA to measure similarity, a metric that we show can produce a score of 1 (*i.e.,* perfect agreement) between nonidentical cluster sets when set 2 contains proteins not found in set 1 (Fig. 2), a situation which is common.

To address some of the limitations of graph-based clustering discussed here, we developed *clust.perturb* (https://github.com/fosterlab/clust-perturb, https://rstacey.shinyapps.io/clust-perturb-tool/), a tool that provides metrics for cluster reproducibility (*repJ*) and the reproducibility of nodes within clusters (*fnode*). Because clusters fragment predictably in response to random noise (Fig. 5), it is possible to represent real, future alterations to the network (for instance, changes to a network across replicates of an experiment), with *in silico* network perturbations (*i.e.,* random rewiring of edges within the network). We found that cluster robustness in response to *in silico* network noise was predictive of clusters that were stable across networks derived from independent experiments (Fig. 8), and conversely, *clust.perturb* identified clusters that were spurious network associations that were not reproduced in subsequent experiments. Therefore, *clust.perturb* can provide additional computational evidence that identifies robust clusters, which are more likely to carry true, real-world meaning (Fig. 7).

Using simulated noise to predict the effects of future network alterations relies on noise being representative of those real-world alterations. In this study, we simulate network noise by the addition or removal of edges between existing nodes, *i.e.,* the same nodes with altered edges. However, some real-world network alterations differ from this, *e.g.,* in the case of subsequent CORUM releases, where much of the network differences result from the addition or removal of nodes (proteins). Future work remains to see how this type of noise (node addition and removal) affects cluster reproducibility.

## Conflict of interest

The authors declare no competing interests.
