# Supplementary material for: On the Robustness of Graph-Based Clustering to Random Network Alterations
Source: Mol Cell Proteomics. 2020 Nov 24;20:100002. doi: 10.1074/mcp.RA120.002275 (PMC7896145; doi:10.1074/mcp.RA120.002275)
Supplement: Supplemental Figures S1–S6 [file mmc1.pdf]

SUPPLEMENTARY FIGURES

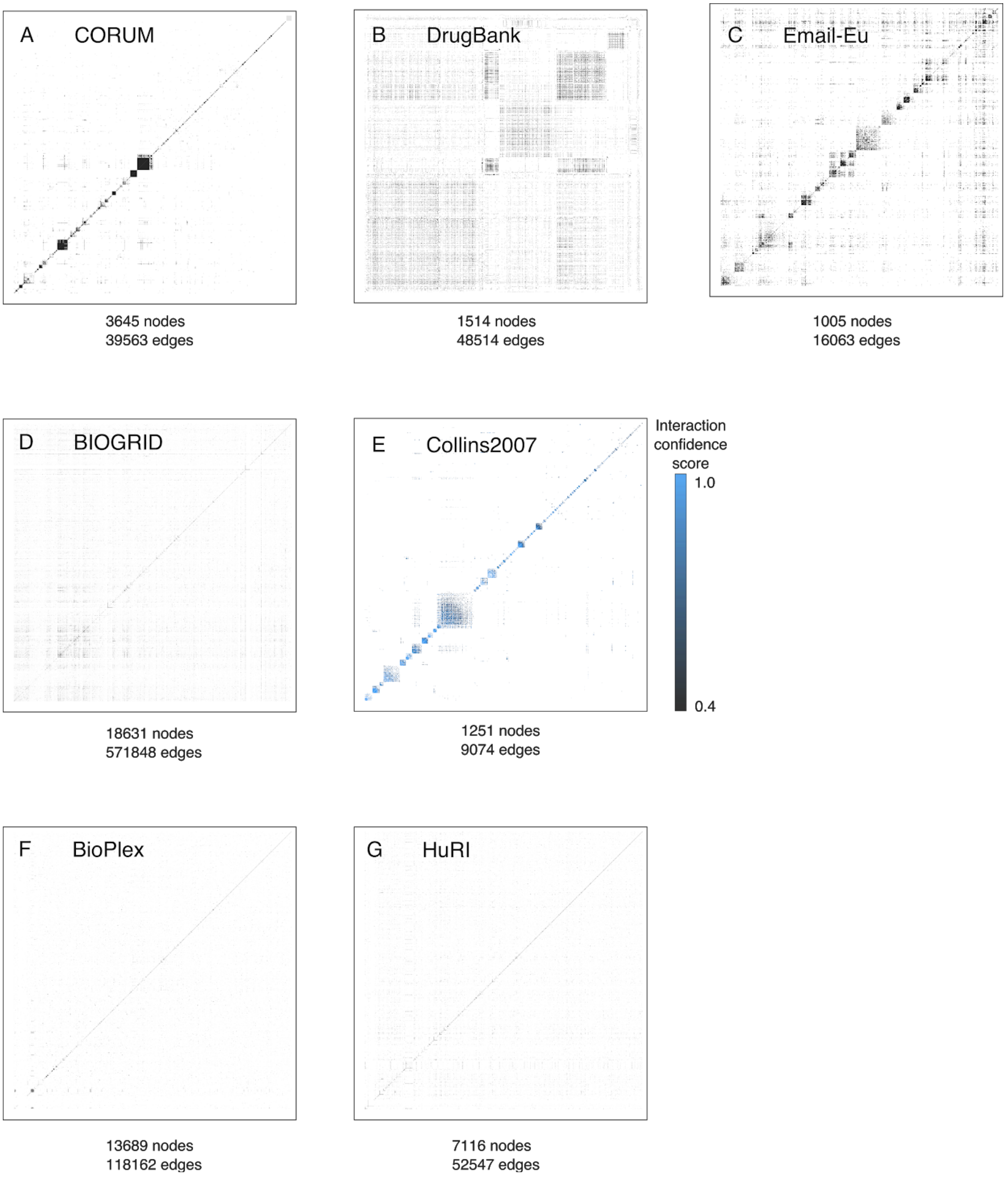

Supplementary Figure 1. **Adjacency matrices of the three network datasets.** A) CORUM network with nodes (proteins) ordered by their first appearance in a CORUM complex. B) DrugBank network. C) email-Eu network with nodes (research institute members) ordered by faculty affiliation. D) BioGRID network. A subset of the full adjacency matrix is shown. E) Collins2007 network, with color showing interaction confidence score. F) BioPlex network. G) HuRI network. Since there is no ground truth cluster set for DrugBANK, BioGRID, Collins2007, BioPlex and HuRI, nodes are ordered using the clusters returned by MCL.

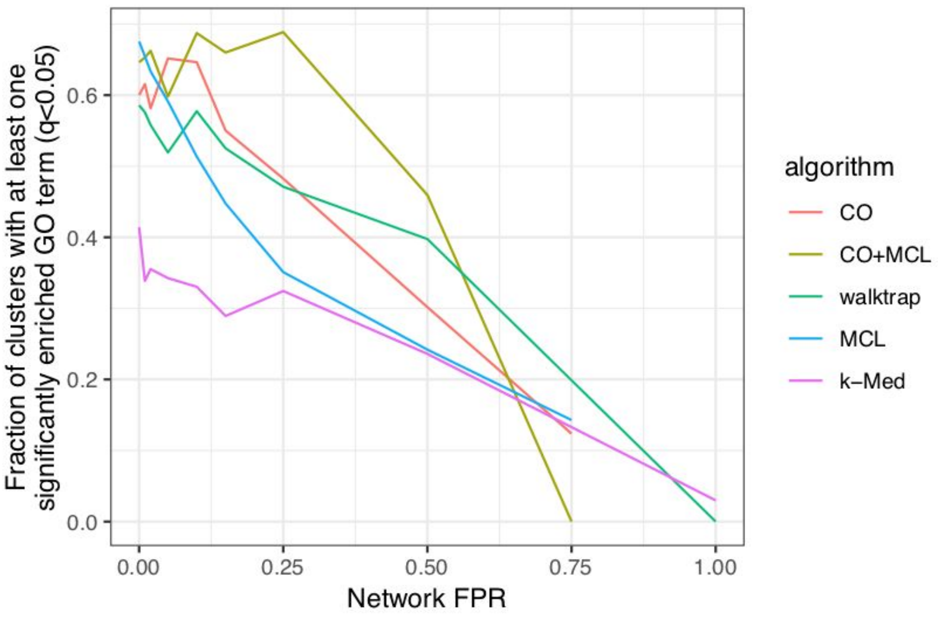

Supplementary Figure 2. **GO enrichment of clustered proteins decreases with network errors.**

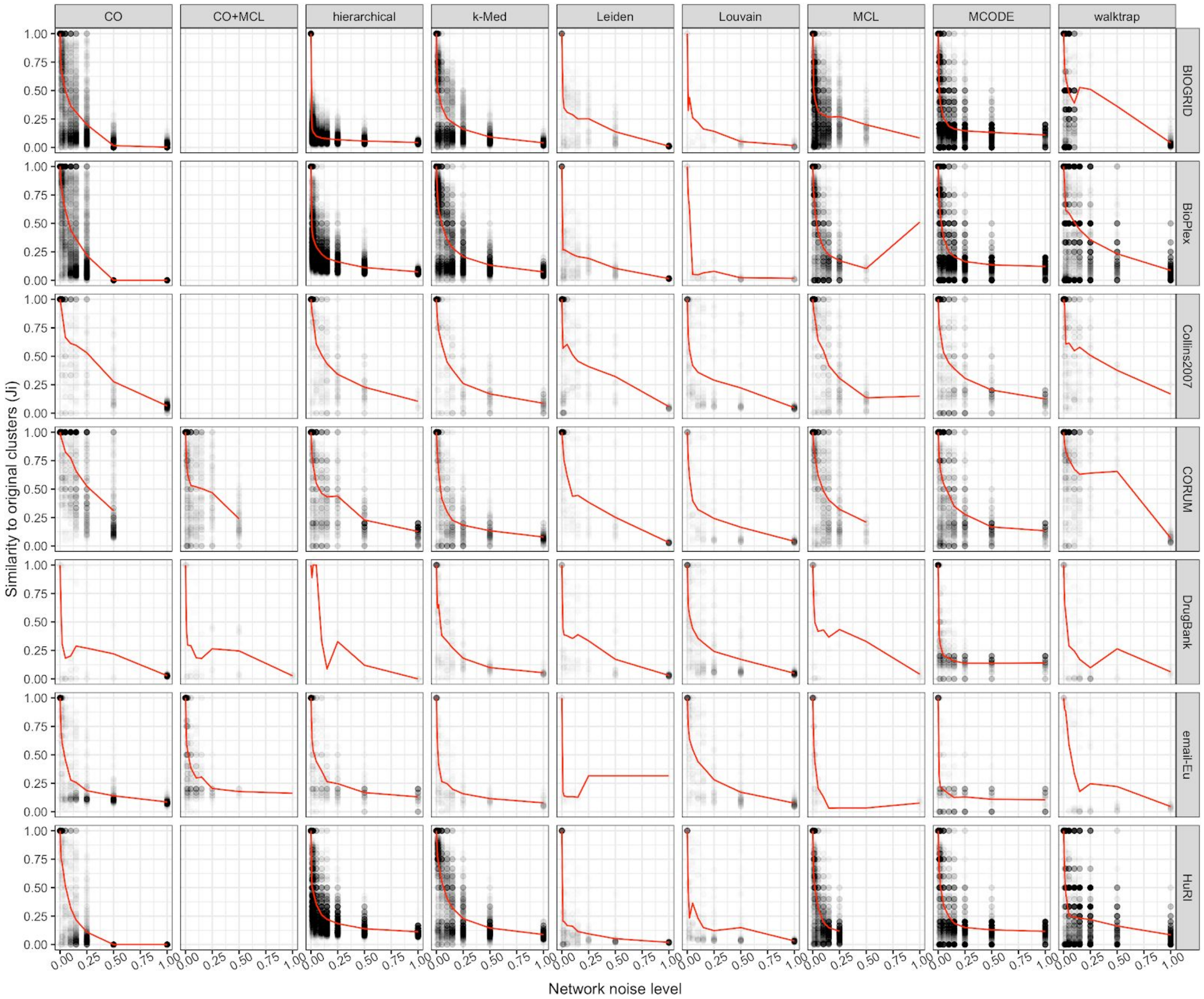

Supplementary Figure 3. **Clustering amplifies network noise.** Full analysis grid of all algorithms and interactomes (not including experiment co-fractionation networks collected by our lab). CO+MCL algorithm was run for a subset of interactomes.

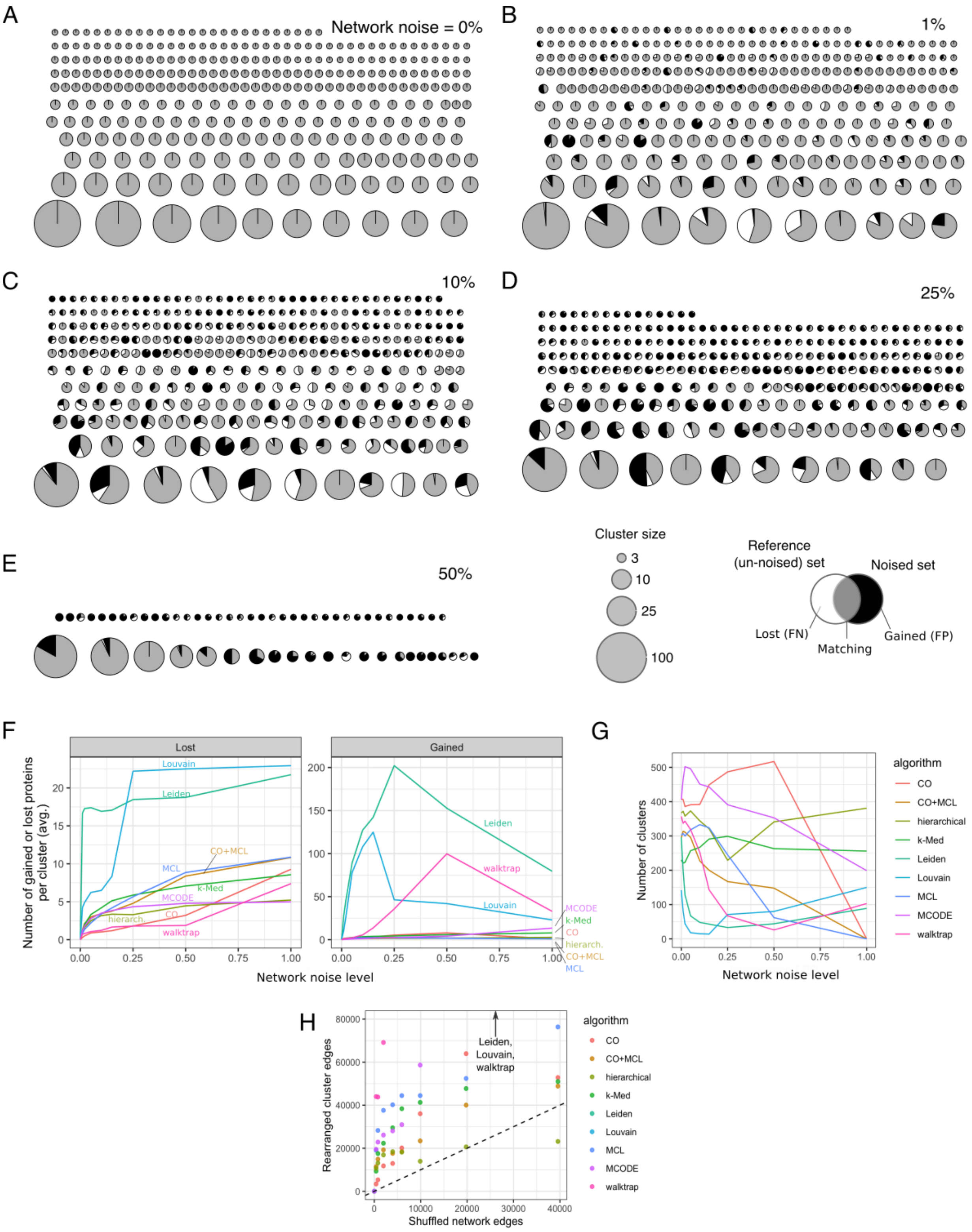

Supplementary Figure 4. **Rearrangement of clustering results in response to interactome noise - visualization and simple counting statistics.** A-E) MCL clustering of the binarized CORUM network after addition of varying levels of interactome noise. Shading shows the agreement with clustering results

from the original network. Grey shows overlap, white shows proteins that are lost after adding interactome noise, and black shows proteins that are added. F) Average numbers of gained and lost proteins per cluster. G) Total number of clusters in each clustering set. H) Comparing the number of altered network edges to the number of rearranged cluster edges.

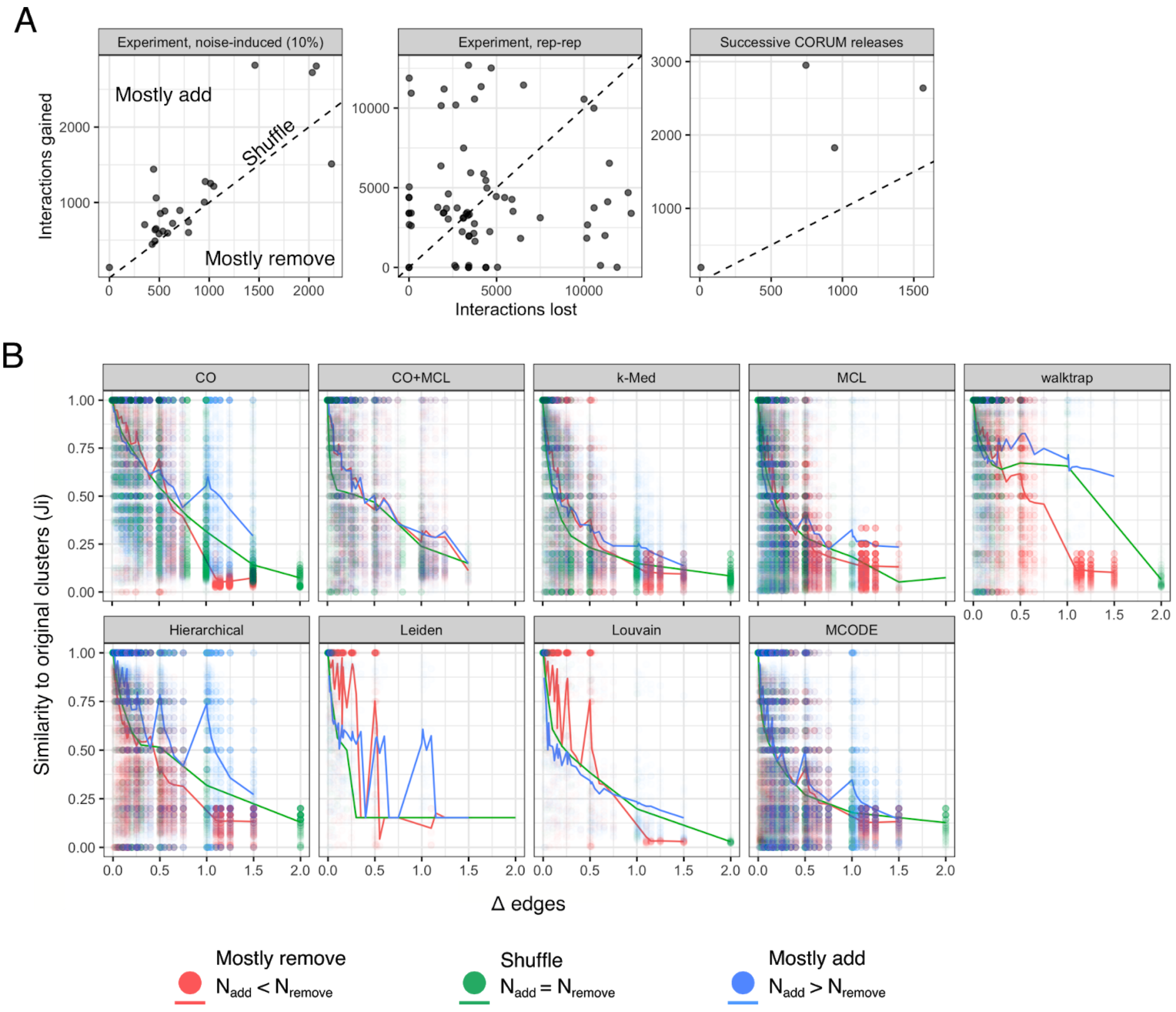

Supplementary Figure 5. **Edge addition and removal, effects on cluster reproducibility**. A) Number of edges lost and gained (x- and y-axis) between different networks. Left: networks from the 28 experimental datasets compared to their networks after adding 10% noise (see Figure 4). Middle: pairs of replicates from the same experiment in the 28 experimental datasets. Right: loss and gain from one CORUM version to the subsequent CORUM version. B) Complementary analysis to Figure 3B, but including all combinations of edge removal and addition. Edges were added or removed in proportion to the original network size (0%, 1%, 2%, 5%, 10%, 15%, 25%, 50%, and 100%). 81 total combinations

(9x9) of edge addition and removal. Δedges is the sum of the added and removed fractions, e.g. 50% removed and 25% added gives Δedges=0.75.

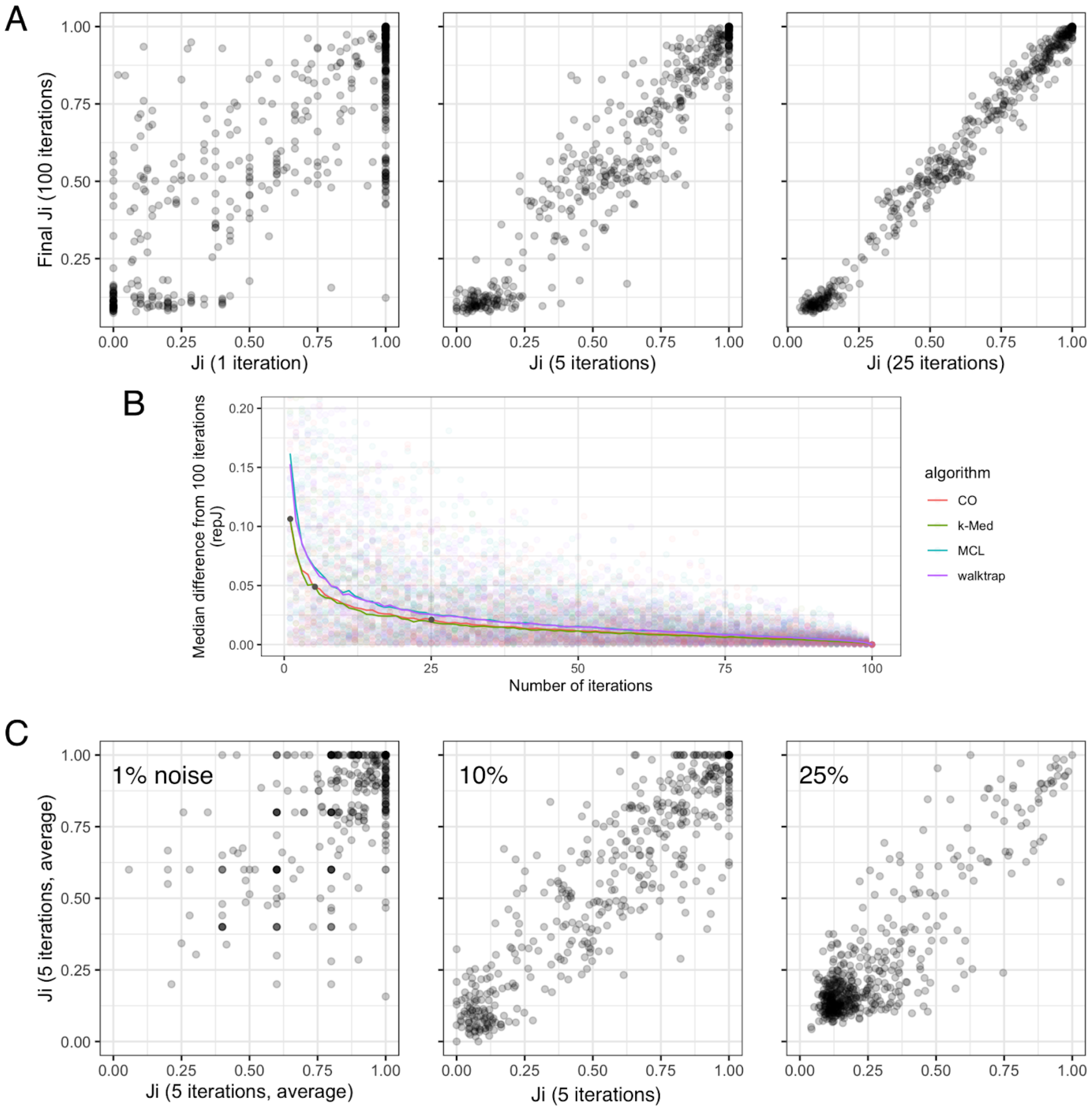

Supplementary Figure 6. **Effects of *clust.perturb* parameters.** A) *Ji* converges towards a final value within few iterations. *Ji* from 1 noise iteration (left), 5 iterations (middle), and 25 iterations (right, x-axis) vs *Ji* from 100 iterations (y-axis). ClusterONE, 10% noise. B) Absolute difference from 100-iteration *Ji* as a function of number of iterations. Panels from A are shown by grey dots. C) Noise level should be chosen to adequately differentiate clusters, as in middle panel (10% noise). Left: too little noise, meaning

most clusters remain unchanged (Ji=1). Right: too much noise, meaning most clusters are disrupted (Ji close to 0).
